# Qualitative and quantitative analysis of diffusion-weighted imaging of gestational trophoblastic disease: Can it predict progression of molar pregnancy to persistent form of disease?

**DOI:** 10.1016/j.ejrad.2016.12.036

**Published:** 2017-03

**Authors:** Sepideh Sefidbakht, Fatemeh Hosseini, Bijan Bijan, Bahareh Hamedi, Tayyebeh Azizi

**Affiliations:** a Medical imaging research center, Department of Radiology and Imaging, Shiraz University of Medical Sciences, Shiraz, Iran; b Abdominal Imaging/MR and Nonvascular Interventional Division, University of California, Davis, CA, USA; c Obstetrics& Gynecology Department, Medical School, Shiraz University of Medical Sciences, Shiraz, Iran

**Keywords:** Gestational trophoblastic disease, Hydatidiform mole, Diffusion-weighted imaging, MRI, Apparent diffusion coefficient

## Abstract

**Purpose:**

To describe the diffusion-weighted imaging (DWI) appearance of gestational trophoblastic disease (GTD) and to determine its apparent diffusion coefficient (ADC) values. To evaluate the feasibility of DWI to predict progression of hydatidiform mole (HM) to persistent disease.

**Methods:**

During a period of 6 months, women with preliminary diagnosis of GTD, based on ultrasound and ßhCG levels, underwent 1.5T MRI (T2 high-resolution and DWI; b values 50, 400, 800; sagittal and perpendicular to the endometrium; and T1, T2 Turbo Spin Echo [TSE] axial images). Patients were followed for 6–12 months to monitor progression to persistent form of the disease. ADC values and image characteristics were compared between HM and persistent neoplasia and between GTD and non-molar pregnancy using Mann–Whitney *U* and Fisher’s exact tests, respectively.

**Results:**

Among the 23 studied patients, 19 (83%) were classified as molar and 4 (17%) as non-molar, based on pathology reports. After 6–12 months of follow-up, 5 (26%) cases progressed to persistent disease and 14 (74%) cases were benign HM. There was no significant difference between ADC values for HM (1.93 ± 0.33 × 10^−3^ mm^2^/s) and persistent neoplasia (2.03 ± 0.28 × 10^−3^ mm^2^/s) (P = 0.69). The ADC of non-molar pregnancies was (0.96 ± 0.46 × 10^−3^ mm^2^/s), which was significantly different from GTD (1.96  ± 0.32 × 10^−3^ mm^2^/s) (P = 0.001). Heterogeneous snowstorm appearance, focal intratumoral hemorrhage, myometrial contraction, and prominent myometrial vascularity were more common in GTD compared to non-molar pregnancy (P < 0.05).

**Conclusion:**

Heterogeneous snowstorm appearance, focal intratumoral hemorrhage, myometrial contraction, and prominent myometrial vascularity are among the imaging characteristics of GTD. We cannot use ADC values to predict progression to persistent disease.

## Highlights

• The incidence of GTD in Iran is significantly higher than America and Europe.
• ADC value of GTD is (1.96 ± 0.32 × 10^−3^ mm^2^/s).
• GTD in T1 and T2-weighted images shows heterogeneous “snow-storm” appearance.
• Focal intratumoral hemorrhage is bright in DWI and low signal in the ADC map.
• ADC value and DWI are not helpful to predict progression of HM to persistent disease.

## 1 Introduction

Gestational trophoblastic diseases (GTD) include a spectrum of pregnancy-related diseases caused by abnormal proliferation of the placenta. The spectrum includes both benign hydatidiform mole (HM) and invasive/malignant gestational trophoblastic neoplasia (GTN). GTNs are characterized by a propensity for local invasion and distant metastases. These neoplasms usually follow a molar pregnancy, but can also occur after a normal pregnancy or abortion [1], [2].

The incidence of GTD varies widely in different geographical regions. The incidence of HM in North America, Australia, and Europe is 0.57–1.1 per 1000 pregnancies. In South East Asia and Japan, the incidence is as high as 2 per 1000 pregnancies, and 8 per 1000 pregnancies in Thailand. Epidemiological studies in Iran have reported an incidence of 5.4 per 1000 pregnancies for HM [1], [3], [4].

The potential diagnosis of HM is often made by ultrasound, but histological examination of evacuated material is essential to confirm the diagnosis. Based on accepted guidelines, close follow-up with serum βhCG monitoring every 1–2 weeks after evacuation of HM is essential to detect invasive mole or choriocarcinoma [1], [2]. The diagnosis of GTN is made on the basis of an elevated serum βhCG plateau or a rising titer over a period of several weeks. If three consecutive tests show normal levels, subsequently, the βhCG level should be determined every 3 months for 6 months [1], [2], [5]. This method, which requires weekly follow-up, is currently the only way to detect persistent disease and a definite diagnosis of GTN cannot be made before a significant time has elapsed.

The initial diagnosis of GTD is usually made before the classic symptoms occur, attributable mainly to the widespread use of routine pregnancy ultrasound studies [2], [6], [7], [8]. A few decades ago, this diagnosis was made using transabdominal hysterography or angiography [9], [10]. CT scans and MRIs have also been employed to evaluate GTD [7]. With its superior contrast resolution, MRI has been used to estimate the depth and extent of myometrial and parametrial invasion [8], [11].

Diffusion-weighted imaging (DWI) has now been integrated into routine abdominal imaging, particularly in oncology. Specifically, DWI has been used in endometrial and cervical cancers to determine the differential diagnosis, the depth of invasion, and the tumor response to therapy [12], [13], [14], [15], [16], [17], [18].

Conventional MRI has been evaluated for diagnosing and determining the depth of myometrial invasion and the extent of parametrial invasion in GTD. However, unlike endometrial and cervical cancers, to the best of our knowledge, DWI findings have not been evaluated in GTD. The primary aim of this study was to determine whether DWI and ADC values (as an indicator of microstructure and cellularity) can predict later progression of HM to GTN. If prediction was feasible, we would be able to eliminate the time and cost of the current method of weekly βhCG monitoring. In addition, we would be able to manage the patient before CT scans or MRIs of the chest, brain, and abdomen and pelvis to particularize the extent of disease. We also aimed to describe the DWI appearance of GTD and to measure ADC values of the tumor.

## 2 Patients and methods

### 2.1 Patient population

The institutional ethical committee approved the study, and written, informed consent was obtained from all patients. The study was performed in two teaching hospitals affiliated with Shiraz University of Medical Sciences, namely, Zeinabieh Hospital, which is a fetal-maternal hospital, and Shahid Faghihi Hospital, which is a general hospital. Between November 2011 and May 2012, all pregnant women with early pregnancy bleeding, increased serum βhCG levels, and ultrasound findings suggestive of molar pregnancy were included in the study. Patients who were considered hemodynamically unstable, those with general contraindications to MRI, and patients who did not provide consent were excluded from the study.

### 2.2 Imaging protocol

All patients underwent pelvic MRI at Shahid Faghihi Hospital using a 1.5T system (Avanto, Siemens Medical Solutions, Erlangen, Germany) equipped with an 8-channel body coil on the same day of preliminary diagnosis. The patients underwent TSE T1- and T2-weighted axial images of the pelvis. High-resolution T2-weighted images of the uterus were also obtained both axial and perpendicular to the endometrial lining (in sagittal images).

Echo-planar DWI was obtained both axially and perpendicular to the endometrial lining (in sagittal images) with the following parameters: TR/TE = 2600/95 ms [b = 50,400 and 800 s/mm^2^]; bandwidth 1042 Hz/pixel; section thickness 5 mm; intersection gap 1 mm; field of view (197–244x 242–300); matrix (117x 192); number of signal averages 4; and fat saturation as a fat suppression technique. ADC maps were obtained using the software of the MRI unit on a voxel-by-voxel basis using the slope of logarithmic decay for signal intensity in DWI images (b values of 50, 400, and 800 s/mm^2^) against the b value. Average time of the whole exam was 11 min.

### 2.3 Final diagnosis

The uterus was then evacuated in all patients by suction curettage. Those cases with spontaneous, missed abortion, in which the possibility of molar pregnancy was considered initially, based on clinical, ultrasound findings, and lab data, but in whom pathological findings showed normal villi, were classified as non-molar pregnancies.

Patients with molar pregnancy were followed for 6–12 months. Based on weekly serum βhCG levels, their disease was classified as persistent or non-persistent disease at the end of the follow-up period.

### 2.4 Qualitative image analysis

Images were evaluated, in consensus, by two radiologists with five years experience in body MRI. Image interpretation was conducted on an Infinitt (3, 1, 1, 0, 4) Picture Archiving and Communication System (PACS) workstation. Both readers were aware of the preliminary diagnosis of molar pregnancy, but neither pathology reports, nor final follow-up results were known at the time of image evaluation.

T1- and T2-weighted images were evaluated subjectively for the presence or absence of the snowstorm appearance and transient myometrial contractions. Focal myometrial thickenings that were not visible on ultrasound images were called transient contractions. In addition, the endometrial outline (sharp versus irregular) and the endometrial-myometrial junction (partly-seen versus well-seen) were assessed onT2-weighted images. The form and outline of the endometrial cavity and hemorrhage (focal round or oval versus crescent bleeding) were assessed on T1 and DWI images. Engorged and dilated vessels were also evaluated in the myometrium and subjectively classified as prominent versus non-prominent; this was done separately on T2-weighted and DWI images.

### 2.5 Quantitative image analysis

All measurements, as well as the subjective image interpretations, were performed on an Infinitt PACS station. The regions of interest (ROI) were first drawn on the T2-weighted MR images with the subjectively largest visible tumor surface in the sagittal plane, as well as in the plane perpendicular to the endometrial lining. The ROIs were then copied to the ADC maps. The automatically measured average signal intensity was recorded as the ADC value. We did not exclude obvious hemorrhagic parts; therefore, for calculation of the ADC values, the whole endometrial content was included in the manually drawn irregular ROI.

### 2.6 Statistical analysis

Data were analyzed using SPSS software, version 17. Descriptive statistics were assessed. For comparison of ADC values between invasive and non-invasive trophoblastic disease and non-molar pregnancy, the Mann–Whitney *U* test was used. To evaluate the accuracy of ADC values for the differentiation of molar and non-molar pregnancies, ROC curves were used. To compare qualitative values, Fisher’s exact test was used.

## 3 Results

Over a period of 6months, 23 patients with early pregnancy bleeding, typical ultrasound, and a preliminary diagnosis of GTD were included in the study. All patients were women with an average age of 27years (range: 17–42 years). The range of gestational age at the time of admission was 7–15 weeks. Based on pathology reports, there were 19 (83%) patients with molar pregnancy and 4 (17%) with non-molar pregnancy. Mean size of the lesions was 81 mm in molar and 45 mm in non-molar pregnancies. After 6–12 months of follow-up with the measurement of serum βhCG levels, 5 (26%) of the 19 patients progressed to a persistent form of disease and 14 (74%) patients showed decreasing βhCG levels and were classified as having benign HM.

### 3.1 Quantitative image analysis

**Fig. 1:**
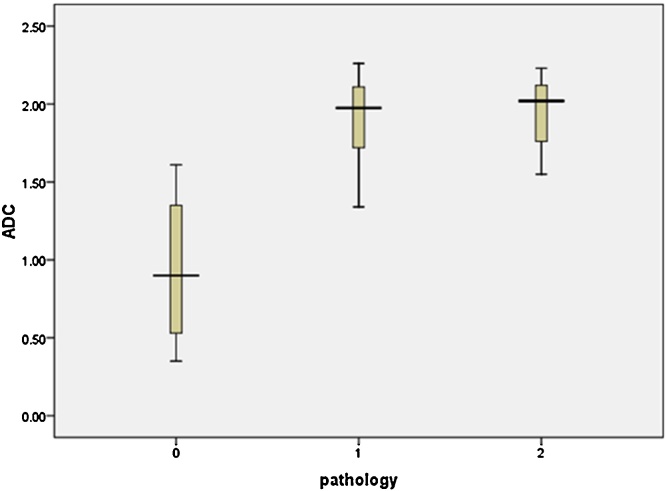
Comparison of ADC values of (0) non-molar pregnancy, (1) benign hydatidiform mole, and (2) persistent trophoblastic neoplasia.

Using the Mann–Whitney *U* test, ADC values were significantly higher in patients with GTD (1.96 ± 0.32 × 10^−3^ mm^2^/s) than in women with non-molar pregnancy (0.96 ± 0.46 × 10^−3^ mm^2^/s) (P = 0.001). ADC values were not significantly different between the benign HM group (1.93 ± 0.33 × 10^−3^ mm^2^/s) and patients with persistent trophoblastic neoplasia (2.03 ± 0.28 × 10^−3^ mm^2^/s) (P = 0.69) (Fig. 1).

**Fig. 2:**
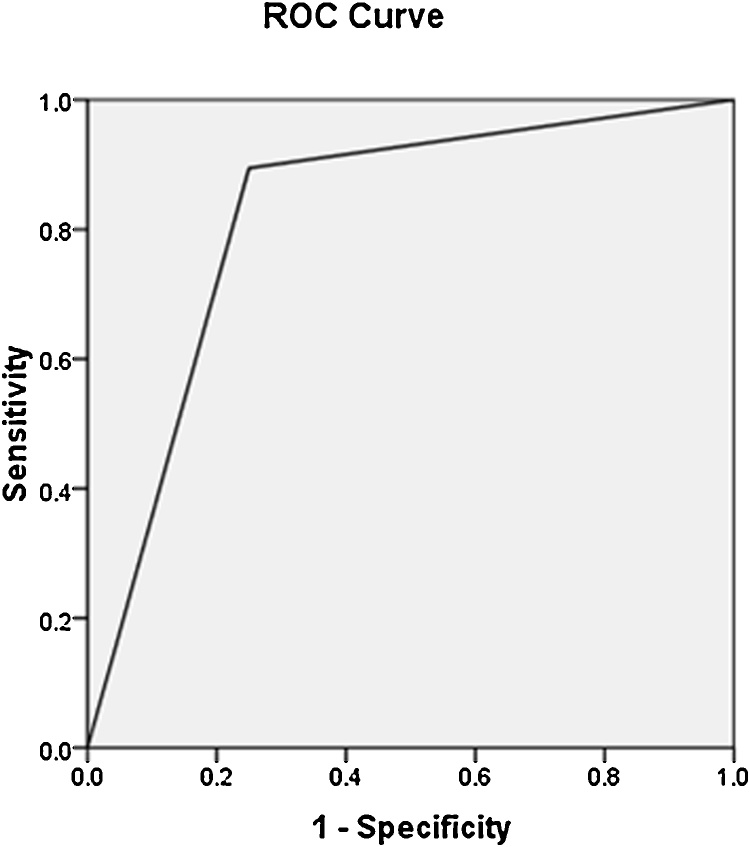
The diagram shows an ROC curve representing the accuracy of ADC values to differentiate molar from non-molar pregnancy.

ROC analysis resulted in a cut-off of 1.5 × 10^−3^ mm^2^/s to differentiate GTD from non-molar pregnancy, with 89% sensitivity and 75% specificity, and the area under the curve was 82 (Fig. 2).

When comparing benign HM and persistent trophoblastic disease, based on a non-significant difference in ADC values, we could not state a cut-off point.

### 3.2 Qualitative image analysis

All patients with GTD displayed a heterogeneous-appearing mass that expanded the endometrial cavity and enlarged the uterus. Expansion of the uterine cavity was associated with significant thinning of the myometrium. In addition, in all patients with GTD, a heterogeneous high signal mass and a snowstorm appearance were seen on T2-weighted images. Focal variable size areas of signal drop were seen, which could have represented acute intratumoral hemorrhage.

**Fig. 3:**
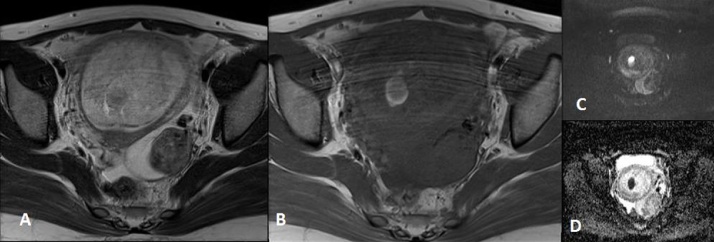
Molar pregnancy in a 24-year-old woman. (A) Perpendicular to the endometrial line T2-weighted MR image of the pelvis showing a snowstorm appearance. (B) Axial T1-weighted MR image showing focal hypersignal for a round and crescent intratumoral hemorrhage, (C) DWI with a b value of 800 showing the same hemorrhage as a bright focus, and (D) an ADC map showing the same hemorrhage as a signal void focus.

OnT1-weighted images, the mass showed an overall heterogeneous low signal and snowstorm appearance, although the mass was slightly hyperintense to the myometrium, with occasional round- or crescent-shaped bright hemorrhagic foci. These hemorrhagic foci demonstrated a high signal on DWI images and a low signal or a signal void on the ADC maps (Fig. 3).

**Fig. 4:**
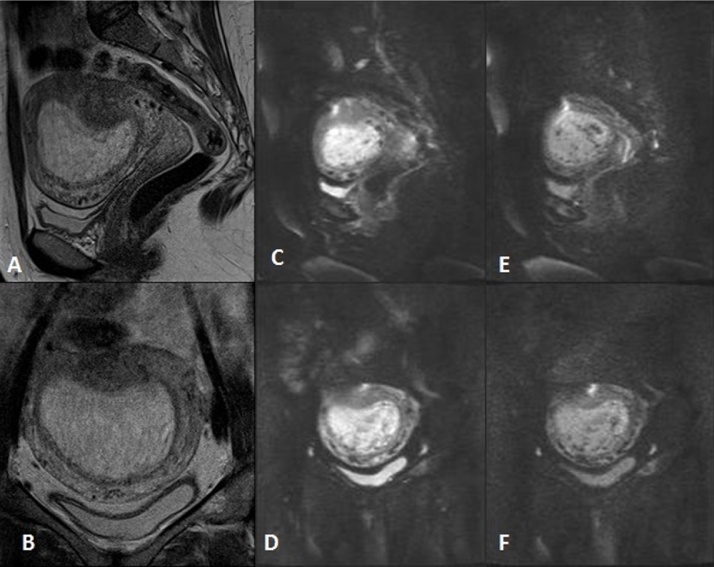
A 30-year-old woman with a molar pregnancy. (A) Sagittal and (B) perpendicular to the endometrial line, T2-weighted MR images show a snowstorm appearance and focal, low-signal myometrial thickening due to contraction. The endometrial line is sharply preserved (C, E) sagittally and (D, F) perpendicular to the endometrial line on DWI with b values of 800 and 400, respectively, and shows prominent myometrial vascularity.

The endometrial line was sharp in all image sequences. Prominent, tortuous myometrial vessels were seen as subjectively more conspicuous on DWI images than on T2-weighted images (Fig. 4, Table 1). Theca lutein cysts, with mean diameter of 62 mm were seen in 4 of the 19 patients, 3 of whom (75%) progressed to persistent disease.

**Fig. 5:**
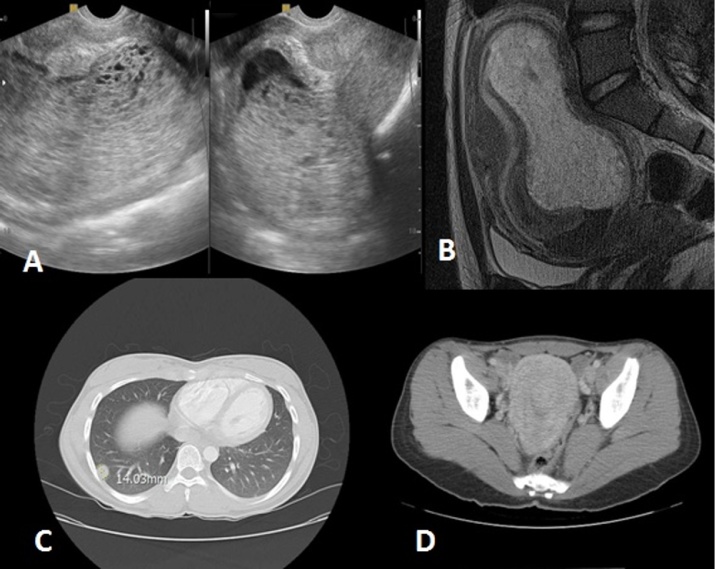
A 21-year-old woman with a molar pregnancy. (A) Sagittal and coronal transvaginal sonography images, and (B) sagittal, T2-weighted pelvic MR images showing a heterogeneous molar mass at the time of preliminary diagnosis. At follow-up, the disease progressed to choriocarcinoma. (C) Axial chest CT scan with a lung window showing metastatic nodules; (D) pelvic CT scan when choriocarcinoma was diagnosed shows a heterogeneous mass in the endometrial cavity.

One of 5 persistent diseases progressed to choriocarcinoma. In this patient’s chest CT scan, multiple small nodules were seen in both lung fields. On the brain MRI, a 12 mm T2 and fluid-attenuated inversion recovery (FLAIR) hypersignal lesion was seen in the right occipital lobe, but Magnetic Resonance Spectroscopy (MRS) images excluded a metastatic lesion. To date, the patient does not report any neurologic problems and her brain MRI was not repeated (Fig. 5).

In patients with non-trophoblastic pregnancy, a snowstorm appearance was described in 4 of 5 patients onT1-weighted images, while a snowstorm appearance was not described in any of the patients in this group onT2-weighted images. The imaging characteristics of a non-molar pregnancy are described in Table 2.

## 4 Discussion

Despite the increasing interest in the functional aspects of MRI, to our knowledge, GTD has seldom been systematically studied with any of the newer functional tools available. This we believe is partly because of its relative rarity, and also because these patients are usually treated with dilatation and curettage urgently upon diagnosis, and obtaining MRI, which is not the standard-of-care in acute settings, is usually not practical.

Among the newer MRI tools, DWI is a versatile and strong imaging modality that can provide unique data regarding tumor cellularity and the integrity of the cell membrane. Reduced ADC values in malignancies occur because of the increased cellular density and the nuclear-to-cytoplasmic ratio in tissues [14], [15], [19].

GTD, which is relatively common in the Middle East, has the unique characteristic of being vesicular and watery, compared to most other tumors, rather than cellular and dense. GTD is classified as an abnormal pregnancy, which consists of vesicular swelling of the placental villi associated with an absent or abnormal fetus/embryo [1]. In some cases, GTD can become invasive and persistent. In our study, of 19 cases with GTD, 5 became persistent and one developed metastases and was diagnosed as a choriocarcinoma. The mean ADC values in patients who did and did not progress to persistent disease were not significantly different. In addition, no cut-off point could be determined between the two groups. Based on this series, we concluded that ADC values cannot be used to predict progression to persistent disease. Using a cut-off point for ADC values, however, can differentiate between molar and non-molar pregnancy.

ADC values have been used to differentiate between malignant and normal tissues or between malignant tissue and benign entities, as in studies such as Naganawa and colleagues' study to differentiate malignant and normal tissue, and Shen et al. study to differentiate benign from malignant processes of the endometrium [16], [20]. Takeuchi et al. showed that there is no evidence that ADC quantification is useful for ovarian lesion characterization, as there is too much overlap between benign and malignant lesions [21].

Consistent with previous studies performed by Nagayama, Alen, and Cheryl, we found a high T2 signal in GTD, with typical heterogeneity described as a “snowstorm appearance”; although Alen and colleagues described the appearance of the mass as a “cluster of grapes” [3], [7], [11]. Also similar to the studies by Alen and colleagues and Brent and colleagues, we found the T1 appearance of the lesion heterogeneous, with a signal slightly stronger than that of the surrounding myometrium [3], [22]. On T1-weighted images, we again described a “snowstorm appearance”.

We found focal areas of intratumoral hemorrhage as crescent- or round/oval-shaped areas of susceptibility, which demonstrated bright signal on DWI and low signal on the ADC map. Neither the crescent nor the round/oval shapes were seen in non-molar pregnancies. This was a reflection of the disorderly array of tissue in the endometrial cavity seen in abortion.

We also found an uninterrupted endometrial outline and an endometrial-myometrial junctional zone in all patients with GTD, including in those that later became persistent. Hricak et al. reported an interrupted endometrial-myometrial boundary [3], [23], [24].

We also found focal myometrial contractions as focal areas of fusiform myometrial thickening without a clear outline, which appeared as a low signal on both T1- and T2-weighted images. This finding was significantly more common in molar pregnancy compared to non-molar pregnancy. This finding also correlates with the larger volume of the uterus in molar pregnancy and might be attributable to that larger volume.

We also evaluated tortuous myometrial vessels in both DWI and T2-weighted images. Although there was no significant difference in the presence or prominence of tortuous myometrial vessels in GTDs between later persistent or limited disease, these vessels were far better visualized on DWI images compared toT2-weighted images. This finding can be attributed to the susceptibility effects of venous blood, which is better seen in EPI compared toTSE images. This fact can be used in the evaluation of MRI of already invasive molar pregnancies for better evaluation of the myometrial vasculature.

### 4.1 Limitations

Our study has several limitations. First, the study population was relatively small, and, to predict the progression of molar pregnancy to persistent disease, a larger number of cases should be studied in the future. Second, despite the fast MRI protocol employed, transferring the patient to the radiology department was difficult because of the relative emergent state of the patients. Third, because large ROIs were chosen, hemorrhagic areas were included in the calculation of ADC values.

## 5 Conclusion

GTD is a watery tumor rather than a cellular tumor, with a relatively high ADC value. It causes uterine cavity expansion and shows a heterogeneous snowstorm appearance, with intratumoral hemorrhage. We cannot use ADC values to predict progression to persistent disease.

## Conflict of interest

None.

## Funding

This research did not receive any specific grant from funding agencies in the public, commercial, or not-for-profit sectors.

## Figures and Tables

**Table 1:** Imaging characteristics in hydatidiform moles *(Fisher's exact test).

	Frequency in benign HM (per 14 patients)	Frequency in persistent trophoblastic disease (per 5 patients)	P*
T2 snowstorm	12	5	1
T1 snowstorm	12	5	1
Round or crescent focal hemorrhage	13	5	1
Sharp endometrial outline	14	5	1
Endometrial-myometrial junctional zone	6 well seen, 9 partly seen	All partly seen	0.530
Transient contractions	13	4	0.386
Significant T2 myometrial vascularity	7	4	0.064
Significant DW myometrial vascularity	13	5	1

**Table 2:** Imaging characteristics of molar and non-molar pregnancies *(Fisher's exact test P < 0.05).

	Frequency in molar pregnancy (per 19 patients)	Frequency in non-molar pregnancy (per 4 patients)	P*
T2 snowstorm	17	0	0.002
T1 snowstorm	17	4	0.002
Focal hemorrhage	18	0	0.001
Irregular endometrial outline	0	3	0.002
Endometrial-myometrial junction was at least partly seen	19	3	0.002
Transient contractions	17	1	0.02
T2 myometrial vascularity	11	2	0.003
DW myometrial vascularity	18	3	0.003
